# Plasma-Derived microRNAs Are Influenced by Acute and Chronic Exercise in Patients With Heart Failure With Reduced Ejection Fraction

**DOI:** 10.3389/fphys.2021.736494

**Published:** 2021-09-27

**Authors:** Isabel Witvrouwen, Andreas B. Gevaert, Nadine Possemiers, Bert Ectors, Tibor Stoop, Inge Goovaerts, Evi Boeren, Wendy Hens, Paul J. Beckers, Anne Vorlat, Hein Heidbuchel, Amaryllis H. Van Craenenbroeck, Emeline M. Van Craenenbroeck

**Affiliations:** ^1^Research Group Cardiovascular Diseases, GENCOR, University of Antwerp, Antwerp, Belgium; ^2^Department of Cardiology, Antwerp University Hospital, Edegem, Belgium; ^3^Cardiac Rehabilitation Centre, Antwerp University Hospital, Edegem, Belgium; ^4^Laboratory of Experimental Medicine and Paediatrics, University of Antwerp, Antwerp, Belgium; ^5^Department of Nephrology, University Hospitals Leuven, Leuven, Belgium

**Keywords:** microRNA, HFrEF—heart failure with reduced ejection fraction, VO_2_peak, peak oxygen uptake, response, exercise training, adaptation

## Abstract

**Background:** Exercise training improves VO_2_peak in heart failure with reduced ejection fraction (HFrEF), but the effect is highly variable as it is dependent on peripheral adaptations. We evaluated changes in plasma-derived miRNAs by acute and chronic exercise to investigate whether these can mechanistically be involved in the variability of exercise-induced adaptations.

**Methods:** Twenty-five male HFrEF patients (left ventricular ejection fraction < 40%, New York Heart Association class ≥ II) participated in a 15-week combined strength and aerobic training program. The effect of training on plasma miRNA levels was compared to 21 male age-matched sedentary HFrEF controls. Additionally, the effect of a single acute exercise bout on plasma miRNA levels was assessed. Levels of 5 miRNAs involved in pathways relevant for exercise adaptation (miR-23a, miR-140, miR-146a, miR-191, and miR-210) were quantified using RT-qPCR and correlated with cardiopulmonary exercise test (CPET), echocardiographic, vascular function, and muscle strength variables.

**Results:** Expression levels of miR-146a decreased with training compared to controls. Acute exercise resulted in a decrease in miR-191 before, but not after training. Baseline miR-23a predicted change in VO_2_peak independent of age and left ventricular ejection fraction (LVEF). Baseline miR-140 was independently correlated with change in load at the respiratory compensation point and change in body mass index, and baseline miR-146a with change in left ventricular mass index.

**Conclusion:** Plasma-derived miRNAs may reflect the underlying mechanisms of exercise-induced adaptation. In HFrEF patients, baseline miR-23a predicted VO_2_peak response to training. Several miRNAs were influenced by acute or repeated exercise. These findings warrant exploration in larger patient populations and further mechanistic *in vitro* studies on their molecular involvement.

## Introduction

Heart failure (HF) is an increasingly prevalent syndrome with substantial mortality and morbidity due to exercise intolerance and dyspnea at exertion (Ponikowski et al., 2016). Apart from pharmacological treatment, exercise training is a successful multisystem approach in patients with heart failure with reduced ejection fraction (HFrEF) as it significantly improves morbidity and quality of life (Ponikowski et al., 2016). However, the individual response to exercise training in terms of peak oxygen consumption (VO_2_peak) is highly variable, with 55% of HF patients showing insufficient increase (Bakker et al., 2018). Importantly, these VO_2_peak non-responders carry an adverse prognosis, independent of other risk factors, and early identification is mandatory (Tabet et al., 2008). The mechanisms driving the variability in response remain incompletely understood, but evidence is pointing toward both genetic and epigenetic regulation (Gevaert et al., 2019; Witvrouwen et al., 2019).

MicroRNAs (miRNAs) are epigenetic modulators of protein coding genes that act at the post-transcriptional level (Peschansky and Wahlestedt, 2014). They are involved in pathways that are relevant for adaptation to exercise, such as changes in skeletal muscle function and angiogenesis, reduction of inflammation and response to hypoxia (Wada et al., 2011; Hecksteden et al., 2016; Welten et al., 2016; Seo et al., 2017; An et al., 2018; Zheng et al., 2018). We recently identified 5 circulating miRNA (miR-23a, miR-140, miR-146a, miR-191, and miR-210), that predicted the training-induced change in VO_2_peak in HFrEF patients. In a bio-informatics analysis of their gene targets, this miRNA panel showed intriguing relations with biological pathways that could be involved in cardiovascular adaptation to exercise, such as vascular endothelial growth factor (VEGF) and mitogen-associated protein kinase (Witvrouwen et al., 2021). Furthermore, these miRNAs have been related to endothelial function and angiogenesis, skeletal muscle mass and function, and inflammatory processes, all relevant to exercise adaptation (Wada et al., 2011; Zhou et al., 2011; Zhu et al., 2016; Seo et al., 2017; Sun et al., 2017; Mitchell et al., 2018; Zheng et al., 2018; Du et al., 2019; Liu et al., 2019; Qiao et al., 2020).

Previously, it has been shown that miR-146a levels at peak exercise are positively related with VO_2_max, and miR-210 was negatively related to VO_2_max in healthy subjects (Baggish et al., 2011; Bye et al., 2013). Both miR-146a and miR-210 have also been associated with the diagnosis of HF (Vegter et al., 2016). Furthermore, circulating miRNA levels are dynamically regulated by acute and chronic exercise. In healthy subjects, some miRNAs are down- or upregulated immediately after an acute exercise bout, and return to resting levels 24 h after an extended-duration acute exercise bout, depending on the tissues of origin or targets affected by exercise (Baggish et al., 2011, 2014; Nielsen et al., 2014). However, whether circulating miRNAs in HFrEF patients are dynamically regulated after a period of exercise training or by an acute exercise bout is currently unknown.

In this prospective cohort study, we aimed to evaluate whether plasma levels of miR-23a, miR-140, miR-146a, miR-191, and miR-210 are influenced by a 15-week exercise training program. In addition, we assessed the effect of an acute exercise bout on plasma miRNA levels, both in the untrained and trained status.

## Materials and Methods

### Patients and Study Design

In this prospective cohort study, consecutive HFrEF patients that were referred for a 15-week supervised combined strength and moderate-intensity aerobic training program to the Cardiac Rehabilitation Centre of the Antwerp University Hospital (ET group) were compared to age-matched HFrEF patients receiving usual care without exercise training (UC group). Randomization into a training and non-training group was considered as non-ethical in view of the strong indication for exercise training in HFrEF (Class IA indication) (Ponikowski et al., 2016). Patients were included when they completed at least 30 of the 45 sessions. The study complied with the Declaration of Helsinki and was approved by the ethics committee of the Antwerp University Hospital. Written informed consent was obtained from all participants.

**FIGURE 1:**
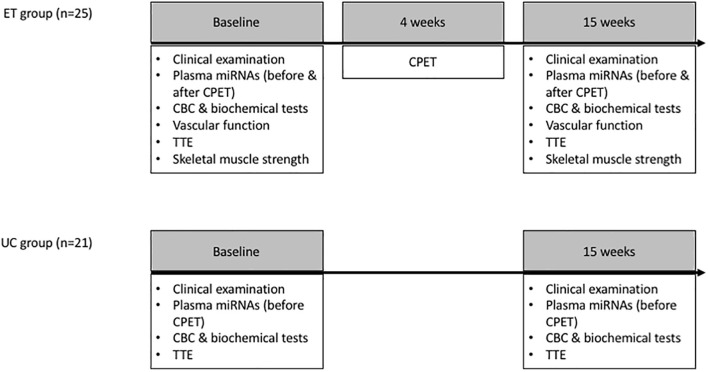
Study design: Plasma miRNA levels were assessed at baseline and after 15 weeks in the ET and UC group. Vascular function, strength characteristics and the effect of an acute exercise bout (CPET) on the miRNA levels were evaluated in the ET group only. Since the relation between miRNAs and the central/peripheral determinants of VO_2_peak (e.g., endothelial function and skeletal muscle strength) was not the primary objective of this study, these determinants were not assessed in the UC group. Vascular function measurements included flow mediated dilation of the brachial artery, pulse wave velocity and heart rate corrected augmentation index. Maximal strength of quadriceps, pectoral, latissimus dorsi, triceps, and deltoid muscles was assessed. CBC, complete blood count; CPET, cardiopulmonary exercise test; ET, exercise training; TTE, transthoracic echocardiography; UC, usual care.

The change in miRNA levels after a 15-week exercise training program was investigated in the ET group and compared the UC group, and baseline miRNA levels were related to the change in VO_2_peak. In the ET group only, the relation between baseline miRNA levels and change in cardiopulmonary exercise test (CPET), cardiac and vascular adaptation, and muscle strength was studied, and the effect of an acute exercise bout on the miRNA panel was assessed (Figure 1).

#### Power Calculation

The sample size was calculated at 20 individuals per group. This offers 80% power to detect a difference in change in VO_2_peak between the 2 groups of 0.9 standard deviations (SD) at a significance level of 5%. Previous studies indicate that the standard deviation of the change in VO_2_peak is typically around 1.4 ml/kg/min (Belardinelli et al., 1995). Hence, a difference of 1.26 ml/kg/min in change in VO_2_peak between the two groups is detectable.

#### In- and Exclusion Criteria

Patients with a left ventricular ejection fraction (LVEF) < 40%, symptoms and signs of HF [New York Heart Association class (NYHA) ≥ II], clinically stable and optimally medically treated for ≥ 6 weeks, aged ≥ 18 and ≤ 80 years were eligible. To avoid the effect of sex-differences in epigenetic regulation, only male patients were included. Exclusion criteria were severe valvular pathology, severe renal failure (eGFR CKD-EPI < 30 ml/min/1.73m^2^), acute coronary syndrome < 4 weeks ago, uncontrolled hypertension or arrhythmias, cognitive impairment, severe pulmonary disease (FEV1 < 60% predicted, severe decrease in diffusion capacity, chronic obstructive pulmonary disease GOLD III-IV), auto-immune disorders, oncologic disease, or inability to exercise.

### Exercise Training

Supervised in-hospital exercise training consisted of combined aerobic and resistance training, 3 sessions/week (58 min/session) for 15 weeks. Aerobic training intensity was set at 90% of heart rate (HR) at the respiratory compensation point (RCP). When RCP was not reached, exercise intensity was calculated using the Karvonen formula [exercise heart rate = rest heart rate + (0.70^∗^ heart rate reserve)] (Karvonen et al., 1957). Strength exercise was an important component of the training program, with the focus primarily on gaining strength during the first 8 weeks. Afterward, aerobic training became more prominent (Supplementary Figure 1).

### Clinical Assessments

CPET was performed on a treadmill (Medical Jaeger, Würzburg, Germany) with a graded protocol (equivalents of 40 W + 20 W/min or 20 W + 10 W/min) (Beckers et al., 2011), with an identical protocol for the follow-up test (Cardiovit CS-200 Ergo-Spiro, Schiller AG, Baar, Switzerland). Gas exchange measurements and 12-lead electrocardiogram were recorded continuously. Blood pressure was measured every minute. VO_2_peak was determined as the mean VO_2_peak during the final 30 s of exercise. Percent predicted VO_2_peak was calculated using the Jones equation (Jones and Campbell, 1982). The RCP was estimated from the systematic increased ventilatory equivalent for VCO_2_ (VE/VCO_2_) and the systematic decrease in end tidal partial pressure of CO_2_ (PETCO_2_) (Whipp et al., 1989; Algul et al., 2017).

Echocardiography was performed on a Vivid E95 cardiac ultrasound using the 4V transducer for 3-D imaging and analyzed on Tomtec Arena). Left ventricular ejection fraction (LVEF), left ventricular mass index (LVMi), left ventricular end diastolic volume (LVEDV), left atrial volume index (LAVi), interventricular septum (IVS) thickness and diastolic parameters (E/A, E/e’) were recorded. In the UC group, LVEF was calculated using Simpson’s monoplane (4 chamber view) method on M5S transducer, AGFA IMPAX Agility 8.1.2, Vivid E95.

Endothelial-dependent vasodilation of the brachial artery was evaluated by flow mediated dilation (FMD) as previously described (ProSound alfa6, Hitachi-Aloka Medical Ltd.) (Van Craenenbroeck et al., 2015c; Mannaerts et al., 2019). FMD was expressed as the percent change in peak vessel diameter from the baseline value [(peak diameter − baseline diameter)/baseline diameter]. Endothelial-independent dilation was calculated accordingly after sublingual administration of nitroglycerine. Arterial stiffness was assessed with carotid-femoral pulse wave velocity (PWV) and pulse wave analysis (PWA) that calculates augmentation index (AIx) and heart rate corrected AIx (AIx75) using SphygmoCor (Atcor Medical), as previously described (Van Craenenbroeck et al., 2015b). All measurements were done in triplicate.

Bioelectrical impedance analysis was performed on an Omron BF306 Body Fat Monitor (Omron Healthcare Co., Ltd., Kyoto) using 2 electrodes (1 handle in each hand) to provide estimates of total lean mass and fat mass.

### Plasma MicroRNA Levels

Whole blood was collected after an overnight fast prior to the CPET in ethylenediaminetetraacetic acid tubes (EDTA). The first 3 ml of blood was discarded to prevent contamination with skin epithelial cells and endothelial cells. Samples were centrifuged within 30 min after collection (1,500 g, 15 min) at room temperature, and plasma was stored at −80°C.

MiR-23a, miR-140, miR-146a, miR-191, and miR-210 were quantified in plasma samples using miRNA RT-qPCR. In brief, plasma samples were thawed on ice and centrifuged for 10 min (4°C, 16,000 g). RNA enriched for small RNAs (including miRNAs) was isolated using the mirVana Paris Kit (Thermo Fisher Scientific). Four hundred microliter 2X Denaturing Solution was added to 400 μl of plasma. RNA was extracted using acid-phenol:chloroform and ethanol. The aliquoted eluate was immediately stored at −20°C. Reverse transcription and preamplification were performed using TaqMan miRNA primers (Thermo Fisher Scientific) and multiplex qPCR was done in a CFX96 thermal cycler (BioRad) as previously described (Van Craenenbroeck et al., 2015a). Raw Cq values were calculated in BioRad CFX manager software v.3.1 using automatic baseline and threshold settings. Cq values that were undetermined or > 35 were removed from the analysis, to minimize statistical confounding by high quantification cycle values. Data were normalized using geNorm and relative miRNA levels were expressed as log(2^–ΔCq*^10^4^) (Gevaert et al., 2018).

### Statistical Data Analysis

Data were analyzed using SPSS 26.0 and R version 3.6.0.

Normality of continuous variables was evaluated using Shapiro-Wilk test. Normally distributed data are expressed as mean ± standard deviation (SD), skewed variables as median and range (1st–3rd quartile). Fisher-exact test was used for comparison of categorical variables, independent samples *T*-test or Mann-Whitney *U*-test for comparison of continuous variables. To assess changes with 15 weeks of ET or with acute exercise, linear mixed models were fitted using time and group or visit as fixed effects and patient ID as random effect, or paired samples *T*-test was used as appropriate.

Correlations were assessed using Pearson correlation analysis. Multiple linear regression analyses adjusting for age and baseline LVEF were performed to assess independent determinants of VO_2_peak. A two-sided *p*-value < 0.05 was considered significant.

## Results

### Baseline Patient Characteristics and MicroRNA Expression

Twenty-five patients were included in the ET group and 21 patients in the UC group. Baseline patient demographics, clinical, pharmacological, CPET characteristics, and circulating miRNA levels are shown in Table 1.

**TABLE 1:** Baseline patient characteristics and training adherence.

	**ET (*n* = 25)**	**UC (*n* = 21)**	***p*-value**
**Clinical characteristics**			
Age (years)	55.6 ± 13.4	60.0 ± 9.4	0.199
Male sex	100%	100%	1.0
BMI (kg/m^2^)	26.3 ± 4.7	29.2 ± 4.7	0.042
Diabetes (n, %)	7 (28%)	1 (5%)	0.055
Arterial hypertension	13 (52%)	8 (38%)	0.346
History of smoking	21 (84%)	13 (62%)	0.089
NYHA class	II = 15 (60%) III = 10 (40%)	II = 17 (81%) III = 4 (19%)	0.124
Ischemic origin of HF	15 (60%)	6 (29%)	0.033
CRT or ICD	ICD = 5 (20%); CRT = 4 (16%)	ICD = 11 (52%); CRT = 4 (19%)	0.022 1.0
Creatinine (mg/dl)	1.25 (0.98–1.54)	1.22 (0.96–1.59)	0.947
eGFR (ml/min/1.73 m^2^)	69.2 ± 27.6	66.6 ± 21.8	0.726
**Echo characteristics**			
LVEF (%)	32.5 (25.0–37.0)	30.0 (22.5–37.0)	0.576
**Pharmacological therapy**			
RAAS blocker	25 (100%)	21 (100%)	1.0
Beta blocker	22 (88%)	19 (90%)	1.0
Aldosteron antagonist	18 (72%)	13 (62%)	0.467
Diuretic	16 (64%)	10 (48%)	0.264
**CPET characteristics**			
Resting heart rate (bpm)	66.0 (60.0–71.5)	63.0 (55.0–71.5)	0.440
Baseline VO_2_peak (ml/kg/min)	21.0 ± 6.3	19.2 ± 5.8	0.321
% Predicted VO_2_peak (%—ml/kg/min)	73.0 ± 20.6	71.4 ± 16.8	0.780
RER	1.19 ± 0.1	1.18 ± 0.1	0.736
Work economy (watt/ml/kg/min)	6.4 ± 1.0	7.3 ± 1.3	0.015
Peak systolic blood pressure (mmHg)	140 ± 31.5	129 ± 37.3	0.289
Peak load (Watt)	133.6 ± 39.9	140.5 ± 46.5	0.592
VE/VCO_2_ slope	35.7 ± 6.8	33.5 ± 7.7	0.296
miRNA expression [log(2^–**Δ**Cq*^10^4^)]			
miR-23a	1.49 ± 0.4	1.23 ± 0.5	0.043
miR-140	2.50 ± 0.2	2.46 ± 0.2	0.432
miR-146a	3.66 ± 0.2	3.62 ± 0.3	0.557
miR-191	3.83 ± 0.2	3.87 ± 0.2	0.519
miR-210	1.48 ± 0.3	1.41 ± 0.3	0.401
**Training adherence**			
Sessions completed (max. 45)	41 (39-43)	NA	NA

*Data are expressed as mean ± SD, as median (1st–3rd quartile) or as number of subjects (%).*

*BMI, body mass index; ET, exercise training; CPET, cardiopulmonary exercise test; CRT, cardiac resynchronization therapy; eGFR, estimated glomerular filtration rate; ICD, implantable cardioverter defibrillator; HF, heart failure; LVEF, left ventricular ejection fraction; RAAS, renin-angiotensin-aldosterone system blockers; n, number of subjects; NA, not applicable; NYHA class, New York Heart Association functional class; RER, respiratory exchange ratio; UC, usual care.*

At baseline, ET and UC were similar with regard to demographics and clinical characteristics, except for BMI, which was higher in UC (*p* = 0.042). Ischemic cardiomyopathy was more common in ET compared to UC (*p* = 0.033), and implantable cardioverter defibrillator (ICD) was less common in ET compared to UC (*p* = 0.022). Pharmacological therapy was comparable between ET and UC. CPET characteristics were similar between groups, except for work economy, which was lower in ET compared to UC group (6.4 vs. 7.3, *p* = 0.015).

Baseline miRNA expression was similar between groups, except for miR-23a which was higher in patients referred for ET compared to CG (*p* = 0.043).

At baseline, better heart (LVEF) and kidney (creatinine) function were associated with higher VO_2_peak (respectively, *r* = 0.303, *p* = 0.043, and *r* = −0.514, *p* < 0.001, Supplementary Figure 2). Patients with lower LVEF had higher miR-210 levels (*r* = −0.321, *p* = 0.032, Supplementary Figure 2) independent from age (β = −9.455, *p* = 0.035, 95%C.I. −18.192, −0.717). None of the other baseline miRNA levels were related with LVEF. No significant correlation was found between baseline miRNA levels and baseline VO_2_peak.

### Exercise Training-Induced Changes in MicroRNA Expression

Changes in aerobic capacity and clinical characteristics after 15 weeks of follow-up are shown in Table 2 and Supplementary Figure 3. Change in VO_2_peak was significantly different between the ET and UC group (+ 0.95 vs. − 0.64 ml/kg/min (difference 1.59, 95% CI 0.06, 3.12, *p* = 0.041). NYHA class, peak load and load at RCP significantly improved in ET. Both ET and UC patients performed a maximal exercise test, evidenced by a high respiratory exchange ratio (RER).

**TABLE 2:** Change in clinical characteristics, CPET variables, echocardiographic findings, skeletal muscle strength, and vascular function after 15 weeks of either exercise training (ET) or usual care (UC).

	**ET (*n* = 25)**		**UC (*n* = 21)**		***p*-value for interaction**
	**Baseline**	**15 weeks**	**Baseline**	**15 weeks**	
BMI	26.3 ± 4.7	27.0 ± 4.7*	29.2 ± 4.7	29.1 ± 4.6	0.006
NYHA class (n, %)	II = 15 (60%), III = 10 (40%)	I = 9 (36%), II = 13 (52%) III = 2 (8%), IV = 1 (4%)*	II = 17 (81%), III = 4 (19%)	I = 1 (5%), II = 14 (67%), III = 6 (28%)	0.002
VO_2_peak (ml/kg/min)	21.0 ± 6.3	21.95 ± 7.5	19.2 ± 5.8	18.56 ± 6.2	0.041
Peak load (Watt)	133.6 ± 39.9	156.4 ± 47.9*	140.5 ± 46.5	143.3 ± 45.3	<0.001
RER	1.19 ± 0.1	1.21 ± 0.1	1.18 ± 0.1	1.18 ± 0.1	0.675
VE/VCO_2_ slope	35.7 ± 6.8	37.2 ± 9.4	33.5 ± 7.7	35.5 ± 9.5	0.751
Load at RCP (Watt)	110.5 ± 38.5	127.6 ± 40.7**	128.8 ± 46.2	113.8 ± 52.6	0.031
VO_2_ at RCP (ml/kg/min)	19.2 ± 6.1	20.1 ± 6.1	18.5 ± 6.7	17.7 ± 7.3	0.370
VO_2_ at 50% of peak load during CPET1 (ml/kg/min)	14.1 ± 4.1	12.9 ± 3.8**	11.6 ± 3.9	12.0 ± 4.7	0.022
LVEF (%)	31.17 ± 7.4	37.15 ± 9.9*			
LVMi (g/m2)	161.32 ± 72.0	135.45 ± 63.0**			
RWT	0.33 ± 0.09	0.32 ± 0.08			
LAVi (ml/m2)	45.36 ± 19.3	42.47 ± 16.7			
IVSd (mm)	10.66 ± 2.3	10.55 ± 2.0			
LVEDV (ml)	194.17 ± 55.9	193.58 ± 54.8			
E/A	1.29 ± 0.8	1.22 ± 0.7			
E/e’ (med)	17.4 ± 8.2	19.1 ± 14.6			
E/e’ (lat)	13.8 ± 8.2	12.7 ± 9.1			
Lean mass (kg)	59.6 ± 8.5	61.5 ± 8.1**			
Bio-impedance (%)	26.0 ± 7.2	26.1 ± 6.9			
Quadriceps (kg)	37.07 ± 18.0	51.20 ± 19.2*			
Latissimus dorsi (kg)	46.25 ± 12.3	54.20 ± 12.3*			
Triceps, pectoral and deltoid muscles (kg)	55.80 ± 14.3	63.95 ± 11.6**			
Pectoral muscles (kg)	28.88 ± 10.9	41.33 ± 10.2*			
PWV (m/s)	7.96 ± 2.0	7.63 ± 1.9			
FMD (%)	4.89 ± 3.2	5.18 ± 2.3			
AIx75 (%)	17.06 ± 13.4	17.5 ± 13.0			

Data are expressed as mean ± SD or as number of subjects (%). *p < 0.001, **p < 0.05.

*AIx75, heart rate corrected augmentation index; BMI, body mass index; CPET, cardiopulmonary exercise test; ET: exercise training; FMD, flow-mediated dilation; IVSd, interventricular septal end diastole; LAVi, left atrial volume index; LVEDV, left ventricular end-diastolic volume; LVEF, left ventricular ejection fraction; LVMi, left ventricular mass index; n, number of subjects; NYHA class, New York Heart Association class; PWV, pulse wave velocity; RCP, respiratory compensation point; RER, respiratory exchange ratio; UC, usual care.*

**FIGURE 2:**
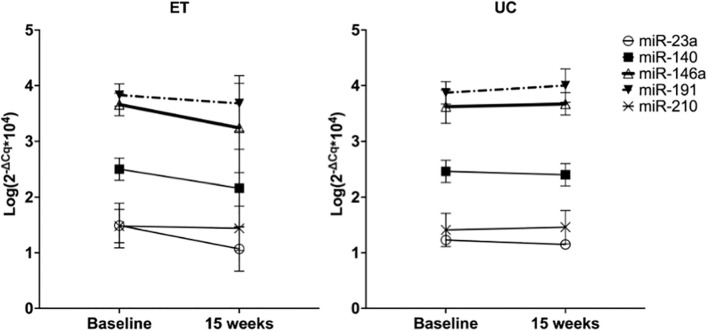
Effect of 15 weeks of training on plasma levels of miRNAs in ET compared to 15 weeks of follow-up in UC. Data are expressed as the mean logarithm of the relative expression of the respective miRNA ± SD at baseline and after 15 weeks. Each line represents the change in plasma miRNA levels with 15 weeks of training in ET (*n* = 25) and 15 weeks of follow-up in UC (*n* = 21).

After 15 weeks of follow-up, plasma levels of miR-146a significantly decreased in the ET group, whereas in the UC group plasma levels remained unaltered (p interaction < 0.05, Figure 2 thick black lines). A significant different evolution in expression levels of miR-191 was observed in ET compared to UC (decrease vs. increase, p interaction < 0.05), but within group differences did not reach significance (dotted-dashed lines, Figure 2). None of the other miRNAs had a significant different evolution between the groups (p-interaction > 0.05).

### Acute Exercise-Induced Changes in MicroRNA Expression

**FIGURE 3:**
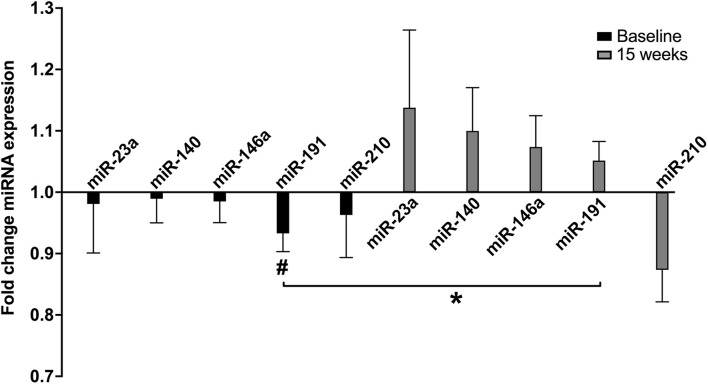
Fold change miRNA expression with acute exercise at baseline and after 15 weeks of exercise training in ET. ET, exercise training group (*n* = 25); Fold change, post CPET/pre CPET miRNA expression. Data are expressed as mean and error. ^#^within group *p* < 0.05, *p-value for interaction < 0.05.

A single exercise bout (CPET) resulted in a rapid and significant decrease in miR-191 levels in untrained HFrEF patients (*p* = 0.043). Intriguingly, exercise training resulted in a blunted and even reversed response to acute exercise (Figure 3); a non-significant increase (*p* = 0.120) after training was observed (p-interaction = 0.003). No significant effect on the other plasma-derived miRNAs was observed, but the same trend of reversal of the miRNA response was observed (except for miR-210).

### MicroRNAs as Predictors for Response to Exercise Training

After 15 weeks of follow-up, VO_2_peak significantly changed in ET compared to UC.

In the ET group only, changes in CPET, echocardiographic, muscle strength and vascular function parameters were assessed as secondary characteristics of adaptation to training. Following training, peak load, load at RCP, VO_2_ at 50% of peak load during CPET1, BMI, LVEF, LVMi, lean mass and strength characteristics significantly improved (see Table 2).

#### Baseline MicroRNAs and Change in VO_2_peak

**FIGURE 4:**
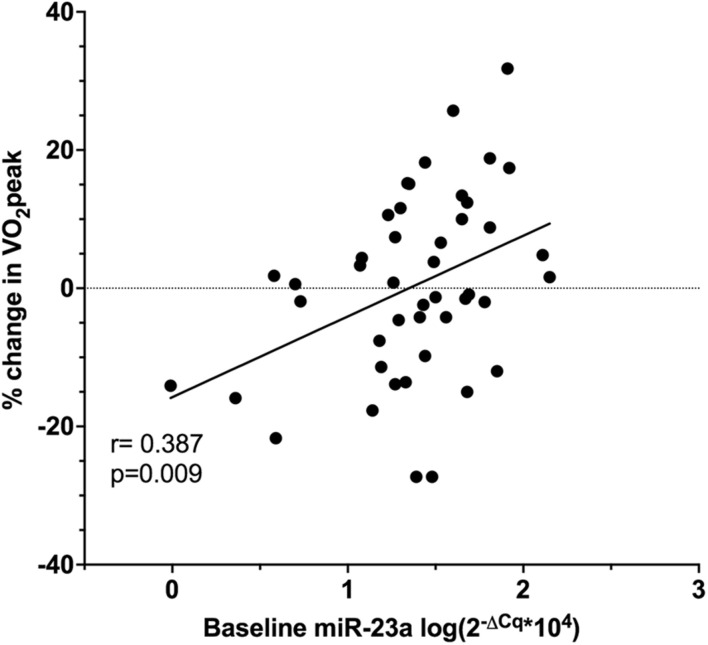
Pearson correlation of baseline relative miR-23a expression and the percent change in VO_2_peak in ET (*n* = 25) and UC (*n* = 20).

Baseline miR-23a was significantly associated with percent change in VO_2_peak (*r* = 0.387, *p* = 0.009, Figure 4), and this was confirmed by multiple linear regression adjusted for age and baseline LVEF (β = 11.307, *p* = 0.017, 95% CI 2.113, 20.500). Other miRNAs were not significantly related to VO_2_peak changes.

#### Baseline MicroRNAs and Training-Induced Changes in Clinical Variables

**FIGURE 5:**
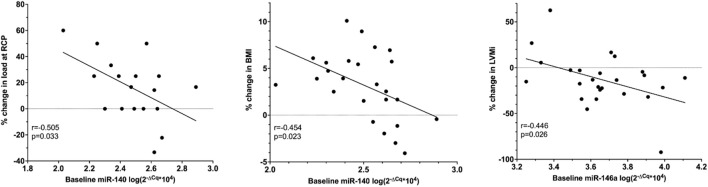
Pearson correlation of baseline relative miRNA expression, the percent change in LVMi, percent change in load at RCP and the percent change in BMI in ET only. BMI, body mass index (*n* = 25); LVMi, left ventricular mass index (*n* = 25); RCP, respiratory compensation point (*n* = 18).

Baseline miR-140 was related with the percent change in load at RCP (*r* = −0.505, *p* = 0.033) as well as the percent change in BMI (*r* = −0.454, *p* = 0.023). Baseline miR-146a correlated with the percent change in LVMi (*r* = −0.446, *p* = 0.026, Figure 5). None of the other baseline miRNAs were related with training-induced changes in clinical variables.

The percent change in BMI, percent change in peak load and percent change in lean mass were not related with the percent change in strength characteristics.

## Discussion

In this prospective cohort study, we investigated the effect of 15 weeks of exercise training as well as an acute exercise bout on plasma miRNA levels in HFrEF patients. Moreover, we studied the relation of miRNA levels with VO_2_peak training response to unravel the underlying mechanisms of adaptation to chronic exercise. The principal findings include:

• miR-146a levels decrease following 15 weeks of training compared to controls
• A single bout of acute exercise results in a decrease in miR-191 in untrained patients
• Baseline miR-23a predicts the percent change in VO_2_peak following 15 weeks of training
• miRNA change in response to exercise may provide insights in the mechanisms driving VO2peak variability.

### Dynamic Regulation of MicroRNA Expression Following Chronic Exercise

As previously reported, expression levels of circulating miRNAs change with acute or chronic exercise training (Baggish et al., 2011; Denham and Prestes, 2016). In the present study, we observed a significant decrease in relative expression of miR-23a, miR-140, and miR-146a in the ET group with 15 weeks of training. However, the evolution was only significantly different for miR-146a when compared to the UC group. Our findings are in contrast with Baggish et al. (2011) who observed no change in miR-146a levels with 90 days of rowing training. This difference might be related to the population studied i.c. athletes. To date, evidence on the physiological role of circulating miRNA in the adaptation to exercise is scarce, and to the best of our knowledge, virtually non-existent in the response to training in HFrEF patients. Hence, we can only speculate that the differences in circulating miRNA levels after training that we observed, may result from an underlying active and selective miRNA process that is involved in pathways relevant to exercise adaptation in HFrEF patients, rather than reduced passive release of these miRNAs.

In HFrEF patients, capillary density in skeletal muscle is reduced (Duscha et al., 1999). Both miR-23a and miR-146a were previously shown to stimulate angiogenesis (Zhou et al., 2011; Zhu et al., 2016). Therefore, reduced miR-23a and miR-146a levels after 15 weeks of training may reflect a diminished need for angiogenesis since capillary density increases with endurance and resistance training (Ingjer, 1979; Hudlicka et al., 1992; Holloway et al., 2018). Also, a transient increase in miR-23a and miR-146a may be expected during the training program, reflecting the exercise-induced angiogenesis, but this needs to be explored in future experiments.

Furthermore, HFrEF patients often have skeletal muscle wasting, especially with more advanced disease status, and this contributes to typical HF symptoms and signs such as dyspnea and exercise intolerance, which results in lower VO_2_peak and load during CPET (Ponikowski et al., 2016). Exercise training improves skeletal muscle mass and function and has beneficial effects on LVEF and LV remodeling in HFrEF patients (Adams et al., 2017; Tucker et al., 2019). An important driver of skeletal muscle wasting is the ubiquitin-proteasome system (Adams et al., 2021). Both miR-23a and miR-140 were shown to protect against skeletal muscle atrophy through inhibiting the ubiquitin-proteasome pathway and Wnt family member 11 expression, respectively (Wada et al., 2011; Liu et al., 2019). Hence, after training, sufficient skeletal muscle hypertrophy may result in lower miR-23a and miR-140 levels. However, this contrasts the finding that baseline miR-140 was inversely correlated with the change in load at RCP and BMI.

In the present study, we observed a differential expression between ET and UC in miR-23a. This could be attributed to the non-randomized study design, where ET patients might have had more skeletal muscle wasting compared to stable sedentary HFrEF controls, as BMI was significantly lower in ET compared to UC. Unfortunately, we do not have strength characteristics of the UC group. After 15 weeks of combined resistance and aerobic training, BMI significantly increased in the ET group, which could be attributed to increases in skeletal muscle mass, as indicated by higher strength characteristics in ER and coinciding increase in lean mass. However, no correlations with strength characteristics, or between (fold change) miR-23a and percent change in strength or lean mass were observed. Regarding the effect on cardiac hypertrophy, both miR-23a, miR-140, and miR-146a mediate cardiac hypertrophy through targeting the ubiquitin-proteasome pathway, GATA binding protein 4 and dihydrolipoyl succinyltransferase, respectively (Wang et al., 2012; Heggermont et al., 2017; Li et al., 2019). In contrast, we observed an inverse correlation between baseline miR-146a and percent change in LVMi in the ET group.

Baseline miR-210 was inversely related to LVEF. Since miR-210 has been related to hypoxia and upregulates VEGF in endothelial cells (Zheng et al., 2018), the inverse relation with LVEF could reflect the reduced oxygen delivery to the periphery that coincides with worsening LVEF and cardiac output in HFrEF (Piepoli et al., 2010).

### Dynamic Regulation of MicroRNA Expression Following Acute Exercise

In addition, miRNA levels can be altered by acute exercise bouts. Previous research in patients with chronic kidney disease showed a rapid downregulation of circulating miR-146a following an acute exercise bout (Van Craenenbroeck et al., 2015a). In patients with heart failure (average LVEF 47.7%), Xu et al. (2016) observed an increase in circulating miR-21, miR-378, and miR-940 with acute exercise. However, in this study no distinction between heart failure with reduced, preserved or mid-range ejection fraction was made. In healthy athletes, miR-146a and miR-222 were shown to be upregulated by acute exercise both before and after a 90-day rowing training, whereas miR-21 and miR-221 were only upregulated by acute exercise before the training period (Baggish et al., 2011). In the present study, at baseline all miRNA tended to decrease following an acute exercise bout, but this was only significant for miR-191. Intriguingly, this response reversed after 15 weeks of ET, which also suggests a selective training-induced effect on the miRNA expression.

MiR-191 has inhibitory effects on angiogenesis in endothelial cells (Gu et al., 2017; Du et al., 2019) and it stimulates myogenesis (Mitchell et al., 2018). As an acute exercise bout in sedentary patients elicits a hypoxic state, this triggers pro-angiogenic mechanisms. The fact that miR-191 has been shown to inhibit angiogenesis therefore could explain the decreased miR-191 levels observed at baseline. However, this needs to be confirmed in *in vitro* experiments. Regarding the effect on myogenesis, a single exercise bout provokes acute muscle damage after which myogenesis is established, and therefore lower levels of miR-191. After this initial decrease in myogenesis, we speculate to observe a rise in miR-191 and stimulation of myogenesis to repair the damaged skeletal muscle cells and to increase skeletal muscle hypertrophy. However, we only collected blood samples immediately after CPET so this hypothesis needs to be confirmed. In addition, increased angiogenesis and reduced myogenesis due to lower circulating miR-191 levels following an acute exercise bout may be conflicting. This could be explained by the fact that miRNA are tissue and disease specific, and circulating miRNA levels not always reflect intracellular levels (Pigati et al., 2010).

Finally, we hypothesize that with repeated acute exercise bouts (i.e., the effect of a 15-week training program) in HFrEF patients, the triggers for angiogenesis and myogenesis might have faded out due to increased capillarity and skeletal muscle mass, resulting in the opposite change of miRNA expression levels.

### Predicting Change in Aerobic Capacity Based on Baseline Plasma MicroRNA Levels

More than half of the HFrEF patients who participate in an ET program may not increase their VO_2_peak (Bakker et al., 2018) and despite many efforts, a predictive biomarker for VO_2_peak response to training is still lacking. In our previous study, we identified several miRNA that were upregulated in patients with an unfavorable VO_2_peak response (Witvrouwen et al., 2021). Among these miRNAs, miR-23a, miR-140, miR-146a, miR-191, and miR-210 were involved in pathways relevant for exercise adaptation processes. In the current study, we observed a significant change in VO_2_peak in ET compared to UC; however, the increase within ET was not significant, which could be explained by the fact that BMI significantly increased in ET. Consequently, the observed change in VO_2_peak in ml/kg/min is underestimated. Furthermore, we confirmed that baseline miR-23a predicts the change in VO_2_peak with training, which may reflect the underlying mechanisms of exercise adaptation since miR-23a was shown to stimulate angiogenesis and to protect against skeletal muscle atrophy (Wada et al., 2011; Zhou et al., 2011). However, we observed clear improvements in muscle strength, but no correlations with miR-23a. This could be attributed to the low sample size. Nevertheless, miRNAs could emerge as promising epigenetic biomarkers of training response.

### Limitations and Future Perspectives

Whereas aerobic training is known to improve endothelial function in stable coronary artery disease and HFrEF patients (Van Craenenbroeck et al., 2010, 2015c), and both aerobic, resistance and combined aerobic/resistance training showed similar improvements in FMD in patients with hypertension or prehypertension (Pedralli et al., 2020), we did not observe significant improvements in vascular function with 15 weeks of ET. This could be attributed to this subgroup analysis lacking statistical power to draw definitive conclusions.

Furthermore, the study can be biased due to the non-randomized design. However, as stated in the methods, randomizing patients to a training and control group would have been unethical in view of the class IA recommendation of ET in HFrEF patients with favorable effects on morbidity, mortality and quality of life (Ponikowski et al., 2016). As findings of this study are hypothesis generating, they should be validated in larger prospective trials and in *in vitro* experiments. Future pre-clinical studies could investigate and compare the expression levels in tissue (skeletal muscle, endothelial cells) to the observed changes in plasma levels. Hence, the contribution of miRNA to exercise adaptation processes can be examined, as either miRNA post-transcriptionally influence gene expression or they can be an exercise-induced epiphenomenon in these tissues (f.ex. exercise-induced skeletal muscle hypertrophy results in an increased release of miRNAs in the circulation). This will aid in further unraveling of the underlying mechanisms of response to acute and chronic exercise.

## Conclusion

The effect of acute and chronic exercise on the expression levels of 5 circulating miRNAs involved in pathways relevant for exercise adaptation (miR-23a, miR-140, miR-146a, miR-191, and miR-210) was investigated in HFrEF patients admitted to a 15-week combined strength and aerobic training program and compared to the sedentary usual care group.

MiR-146a levels decreased following 15 weeks of training compared to the UC group. A single bout of acute exercise resulted in a decrease in miR-191 levels before, but not after training. Baseline miRNA-23a levels were related with the change in VO_2_peak. Furthermore, baseline miR-140 was inversely related to the percent change in load at RCP and BMI, and baseline miR-146a was inversely related to the percent change in LVMi following 15 weeks of training.

Therefore, miR-23a, miR-140, miR-146a, and miR-191 may provide insights in skeletal muscle, cardiac hypertrophy and angiogenic response to exercise in HFrEF patients. These findings warrant further exploration in larger patient populations and in molecular biology set-ups.

## Data Availability Statement

The raw data supporting the conclusions of this article are available from the corresponding author upon request, for non-commercial purposes, without breaching participant confidentiality.

## Ethics Statement

The studies involving human participants were reviewed and approved by the Ethics Committee of the Antwerp University Hospital Drie Eikenstraat 655, 2650 Edegem, Belgium. The patients/participants provided their written informed consent to participate in this study.

## Author Contributions

IW, AG, AVC, and EVC: conceptualization and writing—original draft. IW, NP, BE, TS, IG, WH, and PB: data collection. IW and EB: formal analysis. AG, WH, PB, AV, HH, AVC, and EVC: supervision. All authors contributed to the article and approved the submitted version.

## Conflict of Interest

The authors declare that the research was conducted in the absence of any commercial or financial relationships that could be construed as a potential conflict of interest.

## Publisher’s Note

All claims expressed in this article are solely those of the authors and do not necessarily represent those of their affiliated organizations, or those of the publisher, the editors and the reviewers. Any product that may be evaluated in this article, or claim that may be made by its manufacturer, is not guaranteed or endorsed by the publisher.

## Supplementary Material

Supplementary Figure 1 Training protocol including aerobic exercises (blue) and strength training (yellow). Latissimus dorsi, pectoral, triceps, deltoid, and quadriceps muscles were resistance trained. c-down, cool-down; ex, exercises; w-up, warming-up; reps, repetitions; R, respiratory compensation point at start; RCPi, respiratory compensation point at 4w CPET; RM, repetition maximum.

Supplementary Figure 2 Pearson correlation of baseline LVEF and creatinine with VO_2_peak, and baseline LVEF and relative miR-210 expression in ET (*n* = 25) and UC group (*n* = 21). ET, exercise training; LVEF, left ventricular ejection fraction; UC, usual care.

Supplementary Figure 3 Distribution of percent change in VO_2_peak in all participants (*n* = 46). Gray, non-responders (<6% increase in VO_2_peak); black, responders (≥6% increase in VO_2_peak).

## Abbreviations

Aix: Augmentation index
AIx75: Heart rate corrected AIx
CBC: Complete blood count
CRT: Cardiac resynchronization therapy
ET: Exercise training
FMD: Flow mediated dilation
ICD: Implantable cardioverter defibrillator
IVS: Interventricular septum
IVSd: Interventricular septal end diastole
LAVi: Left atrial volume index
LVEDV: Left ventricular end diastolic volume
LVEF: Left ventricular ejection fraction
LVMi: Left ventricular mass index
PWV: Carotid-femoral pulse wave velocity
RCP: Respiratory compensation point
RER: Respiratory exchange ratio
UC: Usual care.
