# Supplementary material for: Plasma-Derived microRNAs Are Influenced by Acute and Chronic Exercise in Patients With Heart Failure With Reduced Ejection Fraction
Source: Front Physiol. 2021 Sep 27;12:736494. doi: 10.3389/fphys.2021.736494 (PMC8502864; doi:10.3389/fphys.2021.736494)
Supplement: Supplementary Figure 1 — Training protocol including aerobic exercises (blue) and strength training (yellow). Latissimus dorsi, pectoral, triceps, deltoid, and quadriceps muscles were resistance trained. c-down, cool-down; ex, exercises; w-up, warming-up; reps, repetitions; R, respiratory compensation point at start; RCPi, respiratory compensation point at 4w CPET; RM, repetition maximum. [file Data_Sheet_1.docx]

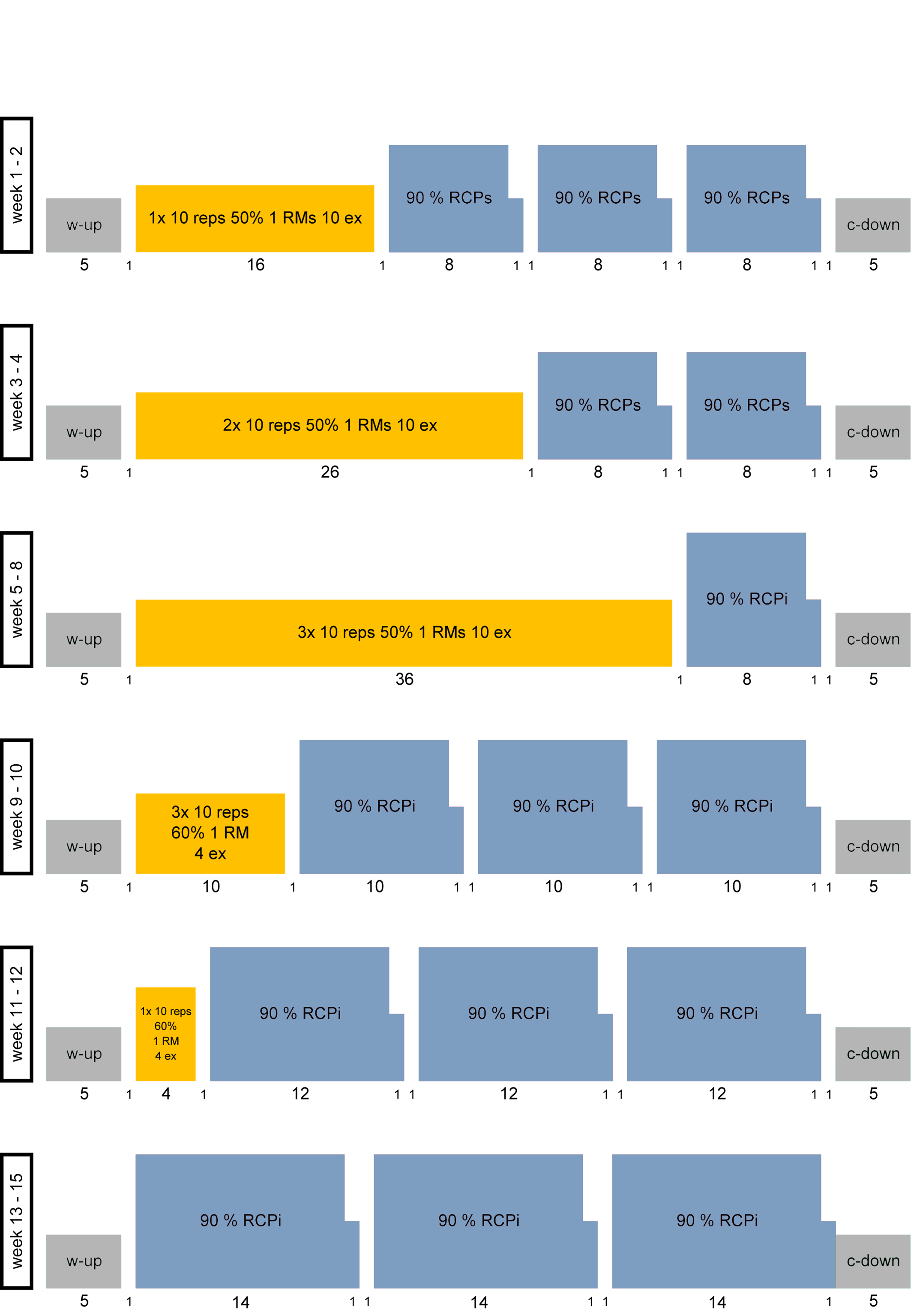


**Figure S1:** Training protocol including aerobic exercises (blue) and strength training (yellow). Latissimus dorsi, pectoral, triceps, deltoid and quadriceps muscles were resistance trained. c-down, cool-down; ex, exercises; w-up, warming-up; reps, repetitions; RCPs, respiratory compensation point at start; RCPi, respiratory compensation point at 4w CPET; RM, repetition maximum.

**
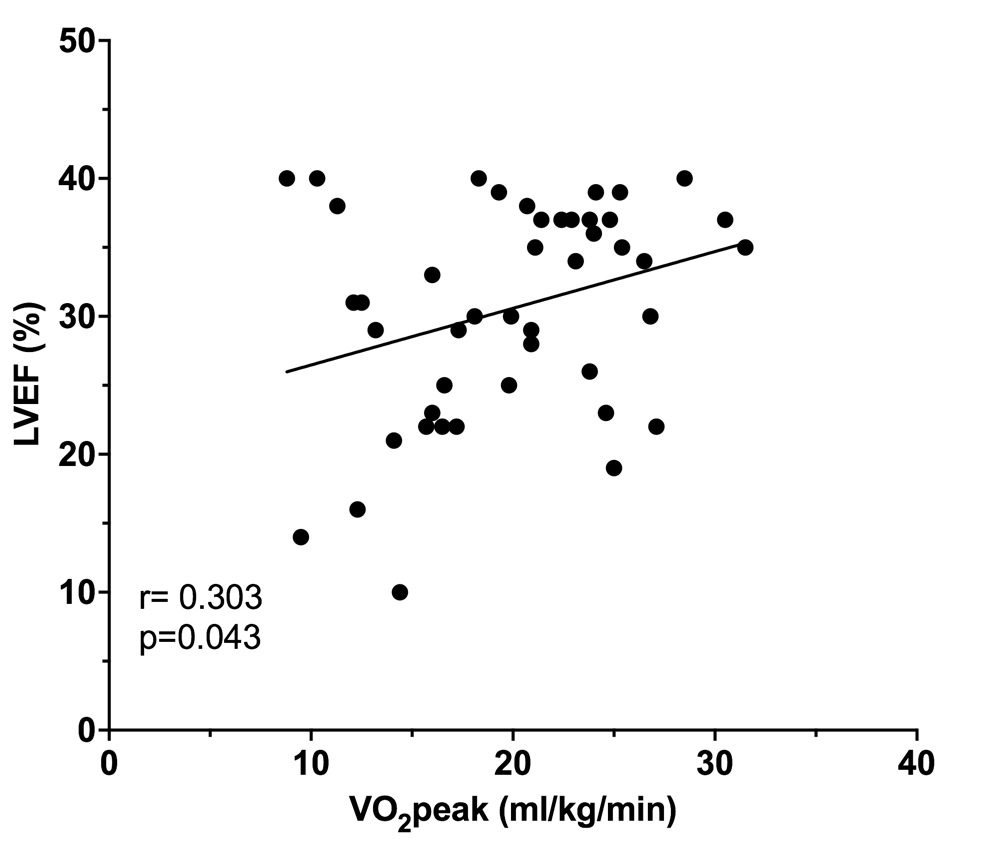

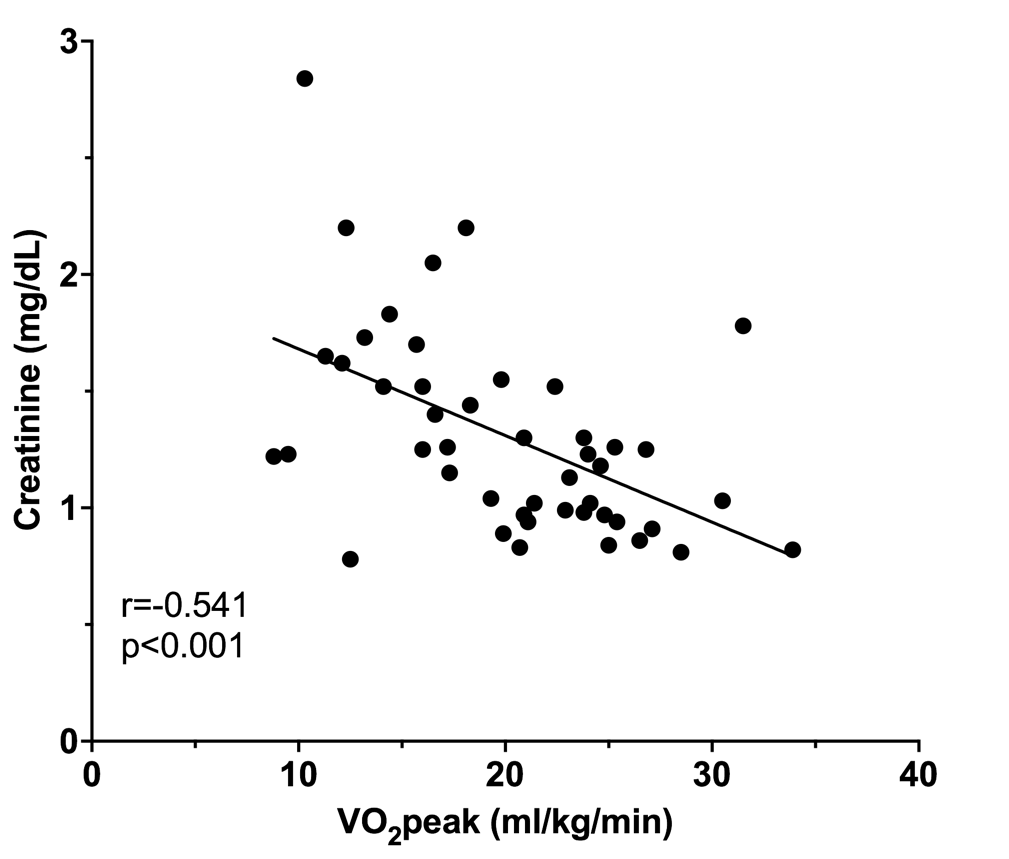

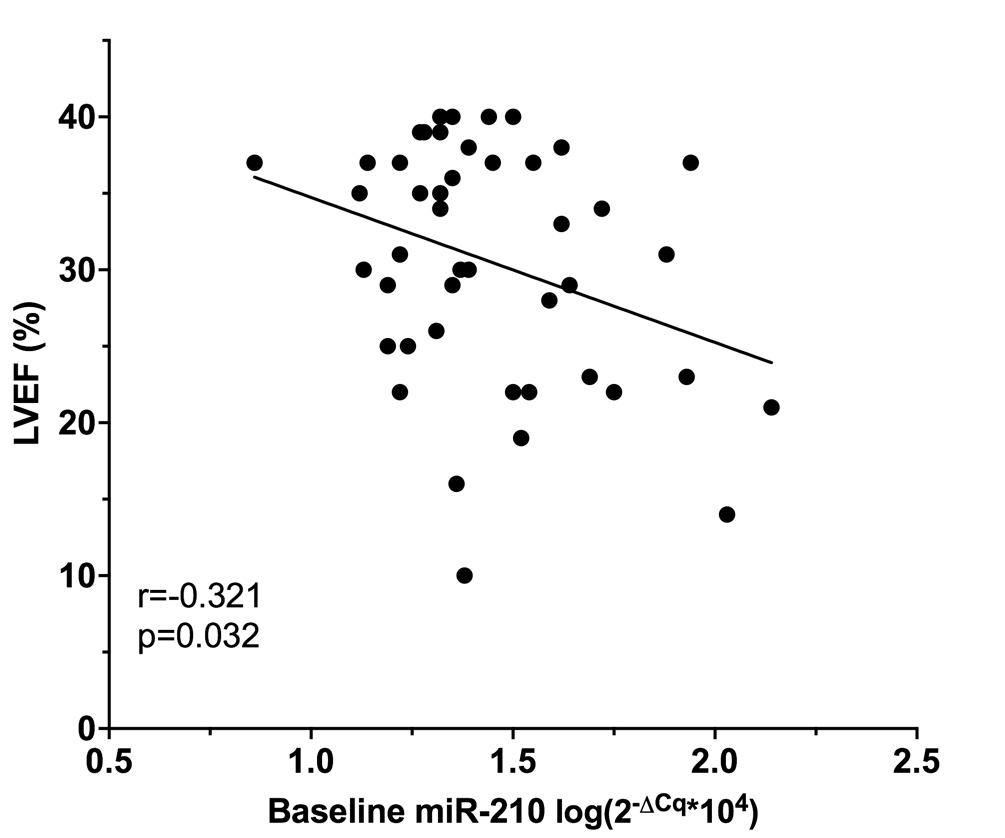
**

**Figure S2: Pearson correlation of baseline LVEF and creatinine with VO_2_peak, and baseline LVEF and relative miR-210 expression in ET and UC group.** ET, exercise training; LVEF, left ventricular ejection fraction; UC, usual care.


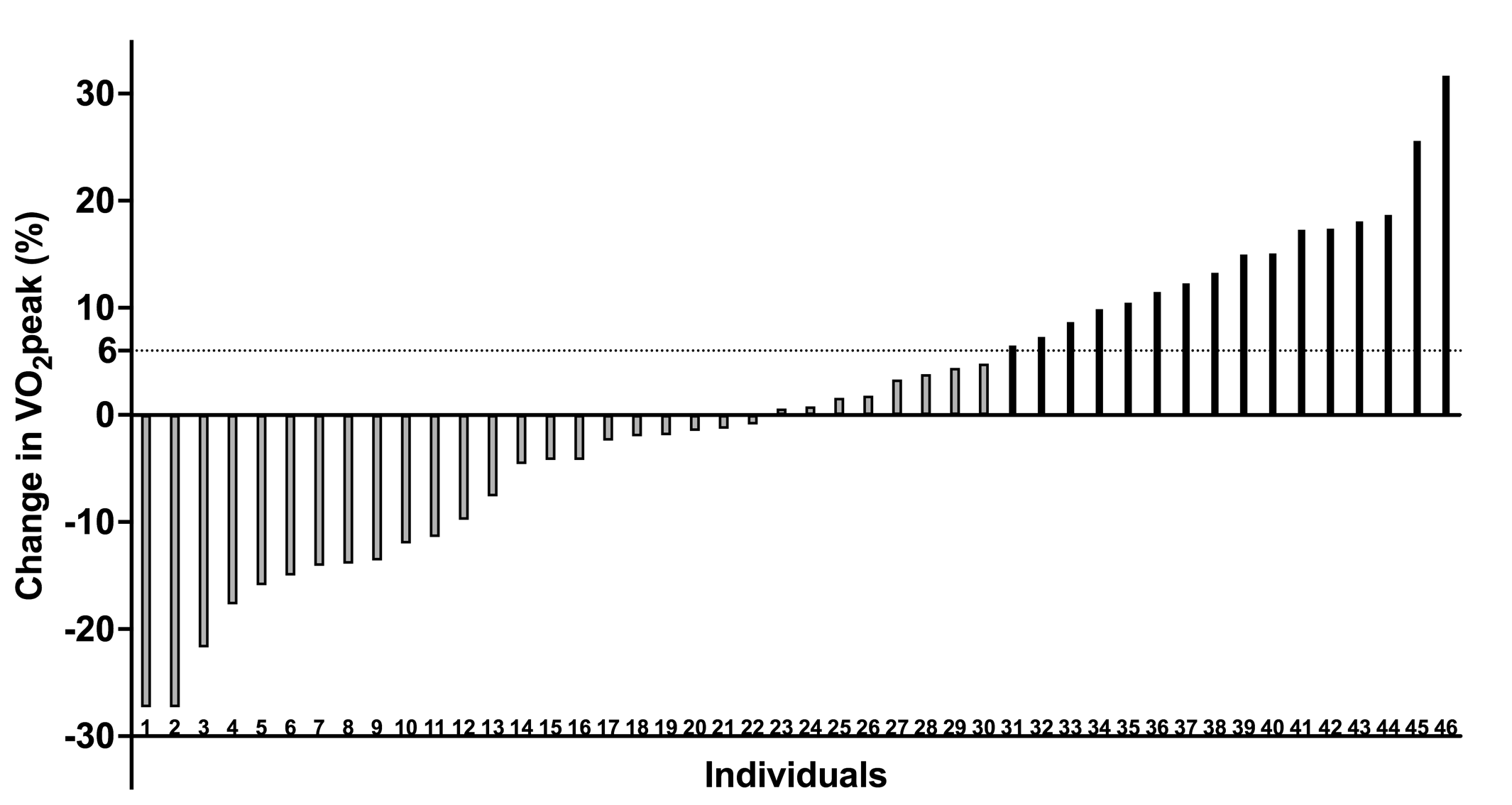


**Figure S3:** Distribution of percent change in VO_2_peak. Grey, non-responders (<6% increase in VO2peak); black, responders (≥6% increase in VO_2_peak).
